# Increased ^18^F-FDG signal recovery from small physiological structures in digital PET/CT and application to the pituitary gland

**DOI:** 10.1038/s41598-019-57313-x

**Published:** 2020-01-15

**Authors:** Marie Meyer, Gilles Allenbach, Marie Nicod Lalonde, Niklaus Schaefer, John O. Prior, Silvano Gnesin

**Affiliations:** 1 Department of Nuclear Medicine and Molecular Imaging, Lausanne University Hospital, Lausanne, Switzerland; 2 Institute of Radiation Physics, Lausanne University Hospital and University of Lausanne, Lausanne, Switzerland

**Keywords:** Molecular medicine, Cancer imaging

## Abstract

On conventional PET/CT, and under physiological conditions, the volume of the pituitary gland (PG) is small, and its metabolic activity is commonly comparable to the surrounding background level in ^18^F-FDG imaging. We compared the physiological ^18^F-FDG uptake of the PG in patients imaged with digital PET (dPET) and with conventional PET (cPET). Additionally, we performed phantom experiments to characterize signal recovery and detectability of small structures. We retrospectively included 10 dPET and 10 cPET patients and measured PG SUVmax, SUVmean and SUVratio (using cerebellum as reference). We imaged a modified NEMA/IEC phantom with both dPET and cPET (background activity 5 kBq/mL, and 3× and 5× higher concentrations in ∅2–20-mm spherical inserts). Mean recovery coefficients (RCmean) and signal-difference-to-noise-ratio (SDNR) were computed to assess lesion detectability. Patients imaged with dPET presented higher PG SUVmax and SUVratio (SUVR) compared to patients imaged with cPET (4.7 ± 2.05 vs. 2.9 ± 0.64, p = 0.004; and 0.62 ± 0.25 vs 0.39 ± 0.09, p = 0.029, respectively), while there was no difference for SUVmean (2.7 ± 1.32 vs 2.1 ± 0.44, p = 0.39). Thus, with a SUV readout scale of 0–5 g/mL, normal PG appeared abnormally hot with dPET, but not with cPET. Phantom evidenced higher RCmean in dPET compared to cPET. For both 3x and 5x measurements, lesion detectability according to size was systematically superior with dPET. In conclusion, patients imaged with dPET presented higher ^18^F-FDG physiological uptake of the PG as compared to patients imaged with cPET. These findings were supported by phantom experiments demonstrating superior signal recovery and small region detectability with dPET. Awareness of this new “higher” SUV of the normal ^18^F-FDG uptake of the PG is important to avoid potential pitfalls in image interpretation, notably in oncologic patients treated with immunotherapy, who are at increased risk to develop hypophysitis.

## Introduction

On conventional PET/CT (cPET), and under physiological conditions, the recovered signal from a small volume structure such as the pituitary gland (PG) is small because its metabolic activity is commonly comparable to the surrounding background level in ^18^F-FDG imaging. Incidental finding of PG hypermetabolism remains rare, and is associated with pathologic lesions in less than half of the cases (pituitary tumour, metastatic malignancy, Langerhans cell histiocytosis or inflammatory hypophysitis)^1,2^. In practice, incidental PG hypermetabolism is a diagnostic dilemma, and its differential diagnosis is challenging, yet, important for clinical decision-making. Patients with PG hypermetabolism should be evaluated for tumour hypersecretion, and patients with macroadenomas should be evaluated for hypopituitarism and other mass effects^3–5^.

It is well known that, the ^18^F-FDG uptake of small structures in PET is underestimated due to the important partial-volume-effects (PVE), affecting the emission signal recovery from these structures^6–8^. Spatial resolution, system sensitivity and image contrast in PET/CT have improved due to recently introduced solid-state digital PET/CT (dPET)^9–11^. A recent study has shown that Siemens Biograph Vision dPET has a 3.7 mm spatial resolution (vs 4.3 mm for Biograph mCT Flow cPET), and a 70.3% increased sensitivity compared to precedent Biograph mCT Flow cPET^11^. Due to improved performances of state-of-the-art dPET compared to cPET, the normal uptake of small structures, such as PG, could result in an improved SUV signal recovery and appear unusually “hot”.

To the best of our knowledge, there are no published data regarding the frequency of incidental PG hypermetabolism detected with dPET, and its clinical significance. The aim of our study was to compare ^18^F-FDG physiological uptake of the PG in patients imaged with dPET and cPET. Additionally, we performed phantom experiments to characterize signal recovery and detectability of small structures in both PET technologies.

## Material and Methods

### Patients population

The procedure followed was in accordance with the ethical standards of the institutional research committee. The local Ethics Research Committee of the State of Vaud approved this research protocol (CER-VD # 2018-101513) and, considering the retrospective nature of the study, waived the need for obtaining patient informed consent. We included 20 consecutive patients who underwent whole-body ^18^F-FDG PET/CT between June and July 2018, for initial staging or follow-up evaluation of their oncologic diseases. None of the patients was known to have a pituitary pathology. Population characteristics are summarized in Table 1.

**Table 1:** Population Characteristics.

	dPET Group (n = 10)	cPET Group (n = 10)	Significance (p)
**Demographic data:**			
Age (years)	60.9 ± 11.8	57 ± 20.4	0.63
Gender (Male/Female)	7/3	6/4	1.00
**Doses of** ^**18**^**F-FDG:**			
Weight (kg)	72.3 ± 17	74.9 ± 20.7	0.91
Activity of ^18^F-FDG (MBq)	153.8 ± 37.3	256.1 ± 54.9	**0.001**
**Type of cancer:**			
ENT	7	5	
Lymphoma	2	—	
Thyroid	1	4	
Melanoma	—	1	
**Prior Treatment:**			
Chemotherapy	3	2	
Radiotherapy	0	0	
Immunothérapy	1	0	

^18^F-FDG: ^18^F-Fluoro-Deoxy-Glucose; MBq: MegaBecquerel; PET: Positron Emission Tomography.

### PET/CT acquisitions, reconstructions and data analysis

All patients fasted for at least 6 h before the PET/CT scan. We imaged 10 patients on a cPET (GE Discovery 690^12^) and 10 patients on a dPET (Siemens Biograph Vision 600^11^), using for both groups, the standard clinical protocol for oncologic acquisitions described here below.

PET images were acquired 1 h after injection of 3.5 MBq/kg of ^18^F-FDG for cPET and 2 MBq/kg of ^18^F-FDG for dPET. Head restraints were applied in order to reduce head movements.

The cPET used a time per bed position of 3 min for the cranio-cervical region, and 2 min for the rest of the body. Acquired data were reconstructed with the vendor software, ordered-subset-expectation-maximization (OSEM) algorithm employing 3 iterations and 16 subsets, an image matrix of 256 × 256 voxels on FOV of 700 mm. The resulting in plane square pixel size was 2.73 mm; the slice thickness was 3.27 mm. A Gaussian post-reconstruction image smoothing (FWHM = 5 mm) was applied.

The dPET Biograph Vision 600 used a flow acquisition of 1.1 mm/s for the cranio-cervical region (equivalent of a time per bed position of 4 min), and 1.4 mm/s for the rest of the body (equivalent of a time per bed position of 3 min). Acquired data were reconstructed with the vendor software, ordered-subset-expectation-maximization (OSEM) algorithm employing 4 iterations and 5 subsets, an image matrix of 440 × 440 voxels on FOV of 780 mm. The resulting in plane square pixel size was 1.65 mm; the slice thickness was 2 mm. Post-reconstruction image smoothing was not applied.

Both PET systems applied time-of-flight information and the point-spread-function correction in the iterative reconstruction process. In addition, all pertinent image corrections (normalization, dead time, activity decay, random coincidence and attenuation and scatter corrections) were applied.

Patient PET/CT data were evaluated by one experienced nuclear medicine physician. All the PET scans were first analysed visually, using a SUV readout scale of 0–5 g/mL We defined PG hypermetabolism as a PG uptake higher than surrounding background. For each patient, we measured the volume (based on CT scan), the SUVmean, and the SUVmax of the PG, and we calculated the SUVratio (SUVR) using the cerebellum as reference region.

### Statistical analysis

All values were expressed as mean ± standard deviation (SD). All data were processed using SPSS. We compared each variable between the two groups using non-parametric test (Mann–Whitney U test and Fisher test). The level of significance was p ≤ 0.05.

### Phantom experiment

A modified NEMA/IEC phantom was imaged in the two PET devices and reconstructed with the same parameters used in clinic. The activity concentration in the background was 5 kBq/mL, a 3×, and 5× higher activity concentration was used in a home-made set of spherical inserts with 2, 3, 5, 7, 10 and 20 mm diameter to simulate condition of different gland to background tracer uptake. The phantom was placed to have the equatorial plane passing through the centre of spherical inserts, at the centre of the PET field-of-view.

In both PET devices and for each phantom background activity concentration, we performed a 5-minute long list-mode acquisition in a step-and-shoot mode. To enable comparison of phantom data, the step-and-shoot acquisition mode was adopted because it was implemented in both PET systems. The continuous bed flow-motion was only available in our dPET system. To characterize the small structure signal recovery and detectability as a function of the acquired signal (proportional to the scan length), we performed additional image reconstructions of 3, 2 and 1 minutes from the original 5 min list-mode dataset. PET reconstructions were obtained using the clinical whole-body ^18^F-FDG PET/CT reconstruction protocols used for patients according to the concerned PET device. CT data were used to draw spherical volume-of-interest (VOIs) on each spherical insert using the PMOD software. In each spherical VOI we measured the average activity concentration (${\bar{A}}_{c,sph,j}$ with j = 1, …, 6). Two cubic VOIs (50-mm side) were placed on the homogeneous phantom background surrounding the sphere to derive the average background value (${\bar{A}}_{c,bg}$) and its standard deviation (σ_bg_).

For both phantom preparations (3× and 5×) and for each spherical insert (j = 1, …, 6), we computed a signal difference to noise ratio (SDNR)

$$SDN{R}_{j}=\frac{{\bar{A}}_{c,sph,j}-{\bar{A}}_{c,bg}}{{\sigma }_{bg}}$$

used to assess lesion detectability (SDNR > 3, according to ROSE criterion).

### Ethical approval

All procedures performed in studies involving human participants were in accordance with the ethical standards of the institutional and/or national research committee and with the 1964 Helsinki declaration and its later amendments or comparable ethical standards. Ethics protocol number: 2018-101513.

### Informed consent

In view of the retrospective nature of the study, the ethics committee waved the need for informed consent.

## Results

### Clinical study

**Figure 1:**
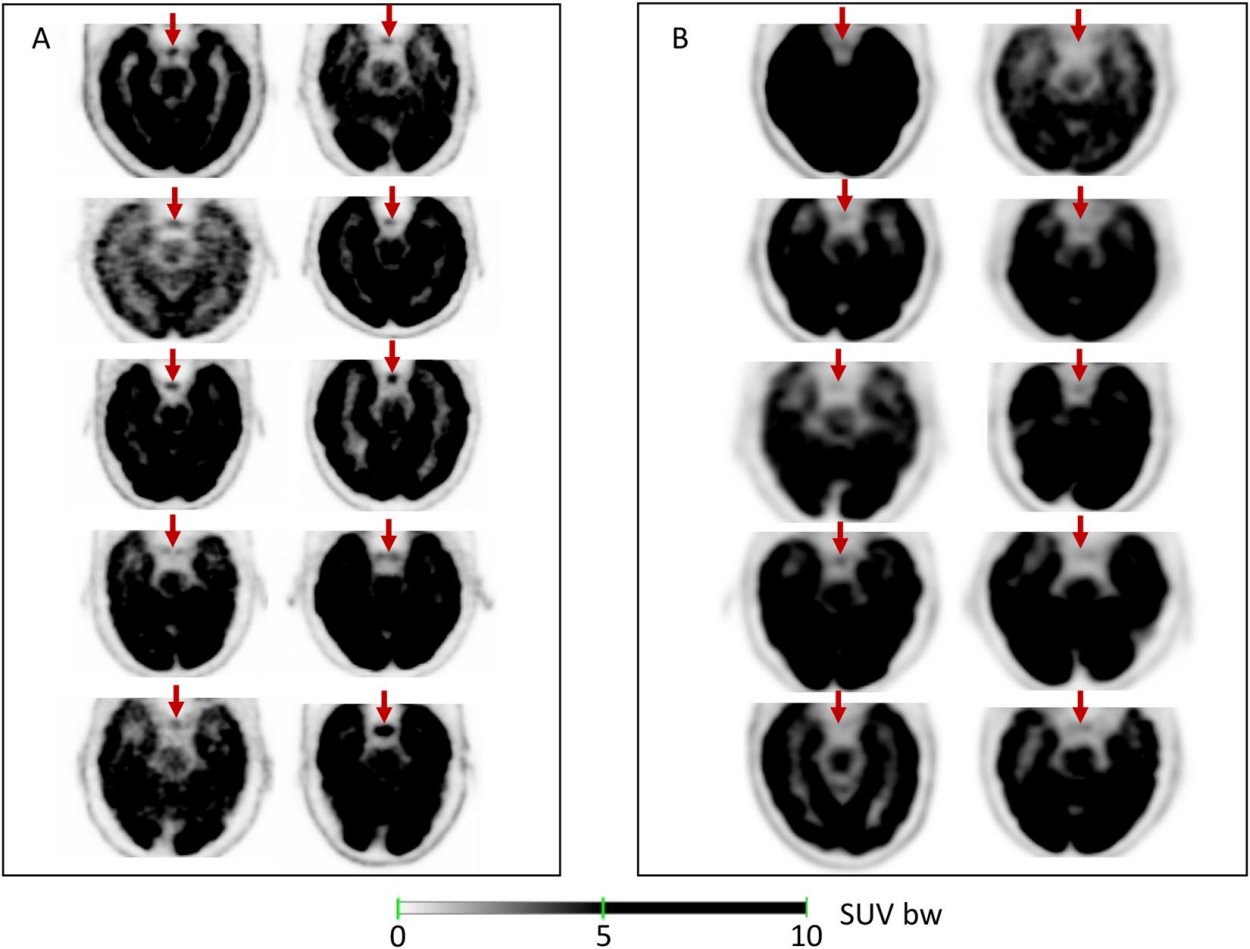
Visual analysis of the signal recovered in Pituitary Gland (red arrows) using a SUV readout scale of 0–5 g/mL in dPET (**A**) and in cPET (**B**).

Concerning visual analysis, 8/10 (80%) dPET patients and only 1/10 (10%) cPEt patients had incidental PG hypermetabolism (uptake higher than background) (Fig. 1).

**Figure 2:**
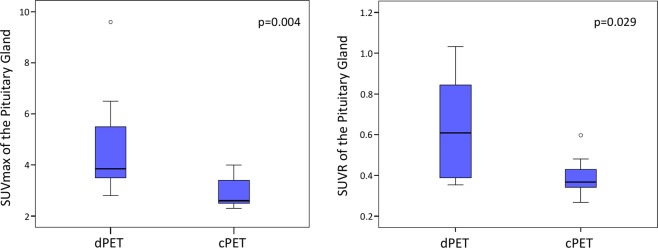
SUVmax and SUVR of the Pituitary gland between the 2 groups of patients.

Concerning quantitative analysis, dPET patients presented significantly higher SUVmax and SUVR as compared to cPET patients (4.7 ± 2.05 vs. 2.9 ± 0.64, p = 0.004; and 0.62 ± 0.25 vs 0.39 ± 0.09, p = 0.029, respectively) (Fig. 2). One dPET patient with very high PG SUVmax (9.6) was further evaluated in order to eliminate PG pathology, and presented a normal MRI without any mass in the PG, and normal blood tests without tumor hypersecretion or hypopituitarism.

**Figure 3:**
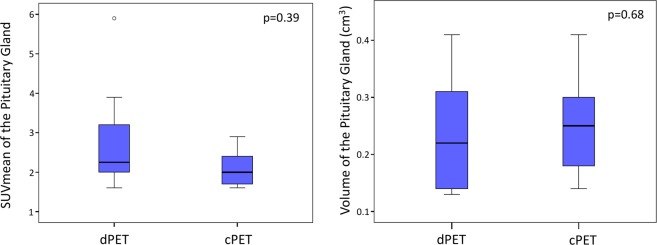
SUVmean and Volume of the Pituitary Gland between the 2 groups of patients.

There was no significant between-group difference concerning SUVmean and volume of PG (2.7 ± 1.32 vs 2.1 ± 0.44, p = 0.39; and 0.24 ± 0.10 on dPET vs 0.26 ± 0.09 on cPET, p = 0.684, respectively) (Fig. 3).

### Phantom experiment

**Figure 4:**
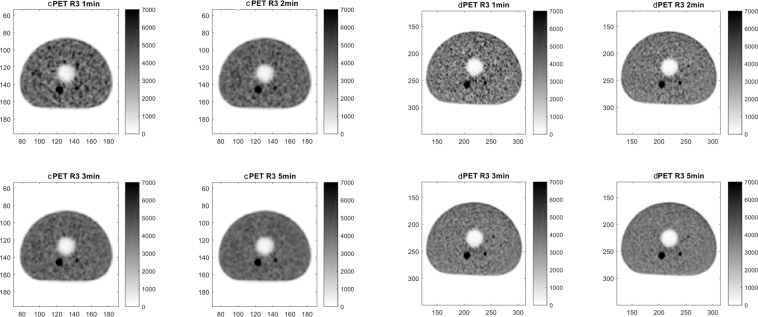
Visual assessment of cPET (left panel) and dPET (right panel) reconstructions for the 3× phantom preparation (activity concentration 3× higher in the spheres as compared to background).

**Figure 5:**
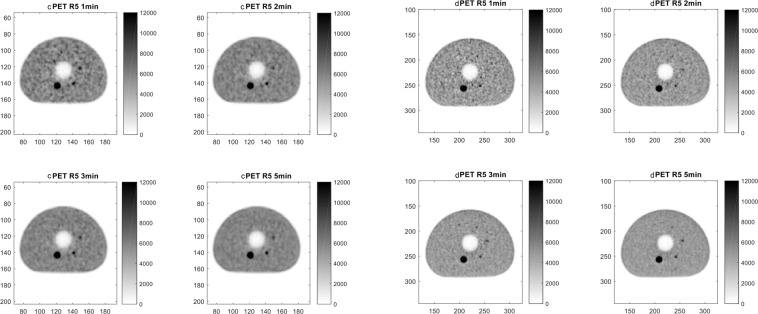
Visual assessment of cPET (left panel) and dPET (right panel) reconstructions for the 5× phantom preparation (activity concentration 5× higher in the spheres as compared to background).

The values of average activity concentration measured in the spherical insert of the phantom with the dPET/CT were higher compared to corresponding values measured with the cPET system. Computed SDNR values for the 3x and 5x phantom preparations are reported in Table 2. In the 3× phantom preparation the smaller detectable insert was of 7 mm (scan time ≥ 3 min) in the dPET and 10 mm (scan time ≥ 2 min) in the cPET. In the 5× phantom preparation the smaller detectable insert was of 5 mm (scan time ≥ 1 min) in the dPET and 7 mm (scan time ≥ 1 min) in the cPET. SDNR values obtained with the dPET, for spherical inserts with diameters ≤ 10 mm, were systematically higher using the conventional PET. Figures 4 and 5 provide a visual assessment of signal recovery in the small structures of the modified NEMA phantom for the 3× and 5× phantom preparations respectively.

**Table 2:** SDNR values for the 3x and 5x phantom preparations.

Sphere (mm)	cPET	dPET	cPET	dPET	cPET	dPET	cPET	dPET
3×	1 min		2 min		3 min		5 min	
20	7.62	7.42	10.33	10.11	13.18	11.95	16.9	15.76
10	**2.82**	3.68	3.13	5.04	4.05	6.36	5.4	8.15
7	**1.2**	**1.49**	**1.15**	**2.7**	**1.74**	3.09	**1.97**	3.03
**5×**	**1 min**		**2 min**		**3 min**		**5 min**	
20	17.55	16.02	23.74	22.25	28.38	27.41	35.67	35.5
10	8.63	10.9	12.09	15.08	12.85	18.2	15.77	23.74
7	5.03	3.33	4.81	5.65	6.04	8.49	6.69	12.34
5	**−0.02**	4.05	**0.5**	3.55	**0.35**	4.37	**0.56**	4.68

PET: Positron Emission Tomography; SDNR: Signal Difference to Noise Ratio. Underlined: SDNR < 3.

## Discussions and Conclusions

In our study, dPET patients presented significantly higher ^18^F-FDG SUV values compared to cPET patients. This improved measured PG signal was not due to an increase in physiological uptake. Instead, it is the result of an increased signal recovery available in dPET compared to cPET, as supported by phantom experiments demonstrating the superior signal recovery and small region detectability achievable in dPET systems compared to cPET.

The study of Van Der Vos *et al*.^13^ outlined the impact of recent advancement in PET on small lesion detection in patients, and used a phantom setup similar to ours. Therefore, reference values according to the PET technology used (hardware, software and its local clinical implementation) are required to distinguish normal from abnormal uptake in small structures (such as the PG or small lesions). PET signal recovery harmonisation, as indicated in the EANM/EARL procedure^14^ can be applied to equalize image quality across different PET technologies and image protocol setups, but for top-performing PET imaging setups this would imply sub-optimal detection and quantitation of the smaller structures.

The improved visibility of small structures with normal or pathological uptake with present state-of-the-art dPET compared to cPET technology results from a combination of different technological improvements. First, dPET is characterized by an optimized match between scintillator crystal elements, semi-conductor amplification and read-out layer with consequent increased spatial resolution and count rate capacity^15^. Second, digital technology contributes to enhance the TOF resolution. In the present dPET generation, TOF has nearly doubled its performance compared to cPET devices (~250 ps in dPET vs. ~500 ps TOF resolution in cPET), with a clear benefit in terms of signal contrast and signal-to-noise ratio^16^. In addition, there is a trend in dPET to extend the axial detector ring extension, thus significantly increasing the systems sensitivity. Interestingly, present developments in PET achieved a total-body coverage^17^. Improved systems sensitivity will be beneficial for patient care in terms of potential reduction of the administered activity (thus resulting in a reduced patient dose exposure) and/or more flexible scan duration increasing patient comfort as recently reported by J. van Sluis *et al*.^18^. In perspective, dPET technology might improve the detection of small lesions not only with ^18^F–FDG, but also with other PET tracers, such as ^68^Ga-PSMA for the detection of lymph node or bone metastases in patients with prostate cancer, or ^68^Ga-DOTA for the detection of small liver or peritoneal metastases in patients with neuro-endocrine tumors. With its improved signal recovery and quantification, dPET will still increase its value in cancer diagnosis and staging, as well as in radiotherapy planning or therapy response monitoring. Finally, the improved signal recovery available in state-of-the-art dPET compared to cPET will probably impact in the future the European Association of Nuclear Medicine (EANM) and EANM Research Ltd. (EARL) guidelines for ^18^F-FDG-PET/CT tumor imaging^13^.

In this study, we have showed that the improvement of signal measurement in dPET leads to an improved physiological uptake in PG. Of course, physiological uptake of other small normal structures (as adrenal glands, ganglia), will be impacted similarly^19,20^.

It is important to understand the differential diagnosis/aetiology of incidental PG hypermetabolism on routine whole body ^18^F-FDG PET/CT. In our study, we have evaluated the ^18^F-FDG physiological uptake of PG. Indeed, it is well known that PG can exhibit physiologic focal hypermetabolism without any underlying lesion or clinical manifestation^1,2^.

On our visual analysis, we defined PG hypermetabolism as a PG uptake higher than surrounding background, as it is the definition most commonly used. We showed that 80% of dPET patients presented PG hypermetabolism, suggesting that using this definition is not adequate with dPET. Moreover, 10% of cPET patient presented PG hypermetabolism. This incidence of PG hypermetabolism is significantly higher than the incidence commonly reported in literature using cPET (<1%)^1,2^. This result can essentially be explain by our small population (10 cPET patients).

We also used semi-quantitative analysis of ^18^F-FDG PG uptake with different parameters (SUVmax, SUVmean and SUVR) and we showed that dPET patients presented significantly higher SUVmax and SUVR than cPET patients. We agree that these values are not universally applicable to all PET centers because SUV is affected by many factors^21–23^. To ou r knowledge, there are no published data regarding quantification of ^18^F-FDG PG uptake with dPET, and there are only a few data with cPET. A previous study has shown that SUV could efficiently differentiate a pathologic lesion from physiologic PG uptake in cPET, with a SUVmax cut-off value of 4.1^2^. In our study, dPET patients presented an average SUVmax of 4.7 (range 2.8–9.6) and none of them were suspected to have PG pathology. It clearly appears that previous cut-off values determined in cPET populations cannot be directly applied to dPET patients.

In our study, we used the CT of the PET/CT for the measure of the volume of PG, although many studies have showed that Magnetic Resonance Imaging (MRI) remains the gold standard. Indeed, MRI allows a more precise analysis of PG structure and a better detectability of small lesions, such as micro-adenoma^24,25^. Moreover, semi-automatic segmentation technique have been developed for MR volumetry of the PG^24^. In our study, we assume that PG of all the patients were normal, and we measure the volume of PG in order to make sure that the difference of SUV between the 2 groups was not due to a difference of volume.

Some limitations of our study can be attributed to the retrospective nature of the presented study design, where only limited data were available on the final diagnosis of these incidental PG hypermetabolic activities. In addition, endocrinologic evaluation and MRI were performed only for one dPET patient with very high SUVmax (9.6) and were entirely normal. Moreover, our population is small (20 patients) which is associated with low statistical power.

At present, there is no defined consensus or published data for the interpretation of focal ^18^F-FDG uptake in the PG. Consequently, we believe our data could serve as a first reference for routine clinical practice. Our study shows that PG hypermetabolism, defined by the “classic” definition based on SUV threshold, presently accepted in conventional PET, is often observed in digital PET and is not necessarily linked to pathological uptake. Awareness of this “new” normal uptake of ^18^F-FDG is necessary to avoid potential pitfalls in image interpretation. This consideration is even more important to keep in mind in the era of immunotherapy, when patients are screened of the appearance of hypophysitis.

## Data Availability

The datasets generated during and/or analysed during the current study are available from the corresponding author on reasonable request.
